# The effects of *in-vitro* pH decrease on the gametogenesis of the red tree coral, *Primnoa pacifica*

**DOI:** 10.1371/journal.pone.0203976

**Published:** 2019-04-18

**Authors:** Ashley M. Rossin, Rhian G. Waller, Robert P. Stone

**Affiliations:** 1 University of Alaska Fairbanks, College of Fisheries and Ocean Sciences, Fairbanks, Alaska, United States of America; 2 University of Maine, School of Marine Sciences, Darling Marine Center, Walpole, Maine, United States of America; 3 Alaska Fisheries Science Center, National Marine Fisheries Services, National Oceanic and Atmospheric Administration, Juneau, Alaska, United States of America; University of Bologna, ITALY

## Abstract

*Primnoa pacifica* is the most ecologically important coral species in the North Pacific Ocean and provides important habitat for commercially important fish and invertebrates. Ocean acidification (OA) is more rapidly increasing in high-latitude seas because anthropogenic CO_2_ uptake is greater in these regions. This is due to the solubility of CO_2_ in cold water and the reduced buffering capacity and low alkalinity of colder waters. *Primnoa pacifica* colonies were cultured for six to nine months in either pH 7.55 (predicted Year 2100 pH levels) or pH 7.75 (Control). Oocyte development and fecundity in females, and spermatocyst stages in males were measured to assess the effects of pH on gametogenesis. Oocyte diameters were 13.6% smaller and fecundities were 30.9% lower in the Year 2100 samples. A higher proportion of vitellogenic oocytes (65%) were also reabsorbed (oosorption) in the Year 2100 treatment. Lower pH appeared to advance the process of spermatogenesis with a higher percentage of later stage sperm compared to Control. There was a laboratory effect observed in all measurement types, however this only significantly affected the analyses of spermatogenesis. Based on the negative effect of acidification on oogenesis and increased rate of oosorption, successful spawning could be unlikely in an acidified ocean. If female gametes were spawned, they are likely to be insufficiently equipped to develop normally, based on the decreased overall size and therefore subsequent limited amount of lipids necessary for successful larval development.

## Introduction

The consequences of anthropogenic activities, including ocean acidification (OA), may have negative effects on primnoid corals and other calcifying organisms [1]. As a result of increased atmospheric CO_2_, average surface ocean pH has decreased by 0.1 pH units since the Industrial Revolution and is projected to decrease by another 0.3–0.4 units by the end of the century [2–5]. The saturation horizons of calcite and aragonite (CSH and ASH, respectively) in the North Pacific Ocean are naturally shallow (~200 m) relative to other oceans [6] and are shoaling at a rate of 1–2 m per year [7]. Recent studies have suggested that calcifying organisms at high latitudes are at immediate risk from OA due to seawater being only slightly supersaturated with regard to calcium carbonate [1,8].

Increased pCO_2_ may have complex effects on the physiology, growth and reproductive success of marine calcifiers, but responses vary among species and populations [9,10]. Under acidified conditions, a greater portion of an organism’s energy budget may be partitioned towards maintenance of the acid-base status of internal fluids and away from other fitness-sustaining processes such as shell and somatic growth, immune response, protein synthesis, behavior, and reproduction [11]. Therefore, OA has the potential to negatively impact sexual reproduction and development of multiple early life stages and may contribute to substantial declines in recruitment that will cascade from community to ecosystem scales [4,12]. OA may impact gamete production by redirecting energy from this non-essential life process to growth, calcification or metabolism maintenance [4,11]. Under stress, some females may reabsorb oocytes to redirect lipids to other cellular processes [13], although this has not been published for cold-water corals.

The primnoidae family is one of the most abundant gorgonian families in deep-sea and polar regions [14] and the most abundant coral family in Alaskan waters [15]. The red tree coral, *Primnoa pacifica* (Kinoshita, 1907), is one of the most common habitat forming and ecologically important cold-water octocorals in the Northeast Pacific Ocean [16–19]. It has a broad geographic range from the Sea of Japan and Sea of Okhotsk, through the Aleutian Islands and Gulf of Alaska (GOA), and to British Columbia and is found in depths from 6–573 m [20]. The shallowest depths recorded are from the GOA where the phenomenon of deep-water emergence occurs in the glacial fjords [21]. These corals are found on habitats dominated by sloping bedrock on rough seabeds and areas with moderate water currents [17].

*P*. *pacifica* exhibits keystone species characteristics [17] as described by King and Beazley [22] and are undoubtedly foundation species [23] in areas of the GOA where they form dense thickets and provide essential habitat for economically important species such as twelve species of rockfish (*Sebastes spp*.) [17,21,24,25]. *P*. *pacifica* is also an ecosystem engineer, modifying its environment by altering small-scale ocean currents and creating living spaces for other organisms [21,26], highlighting this species’ importance to the GOA and shelf fjord ecosystems.

The reproduction and seasonality of a shallow-emerged population of *P*. *pacifica* in Tracy Arm Fjord in southeastern Alaska has been studied by Waller and associates [21]. That study found that an individual colony takes more than 16 months to form mature oocytes (female gametes), which then adhere to the outside of the polyps, where they are most likely fertilized. Each female polyp produces approximately 17 mature oocytes in one cohort, while another cohort of previtellogenic oocytes develops within the mesentery concurrently [21]. Spermatogenesis (production of sperm packets in males) was found to be shorter than oogenesis; oocytes in octocorals tend to develop over a longer period of time than is required for spermatogenesis, as oocytes are energetically expensive to produce [21,27]. Spawning was found to be largely asynchronous with the majority of females maturing in January, whereas most males mature in three cycles throughout a year, from September to December, September to January, and March to June [21].

Some organisms are able to increase internal pH and continue to grow or calcify at normal rates under acidic conditions [28], however, this might come at the cost of affecting other physiological processes, such as reproduction [29]. While corals might physically appear healthy, metabolic costs limiting gametogenesis could be deleterious to the population. Thickets of *P*. *pacifica* are essential to commercial fish and invertebrate species, and as such limiting their range in future oceans would be detrimental to the wider GOA ecosystem. Insights into the possible future population dynamics can be investigated through following spermatogenesis and oogenesis during pCO_2_ manipulations. The objective of this study was to experimentally investigate the effects of OA on overall gamete production of *P*. *pacifica*.

## Materials and methods

Ethical approval for this research was not required by any federal, state, or international law because the animals used were invertebrates. The transportation and field collection of the animals was authorized by the Alaska Department of Fish & Game (Fish Resource Permit CF-16-027). Reference to trade names does not imply endorsement by the National Marine Fisheries Service, NOAA.

### Sample collection area

**Fig 1:**
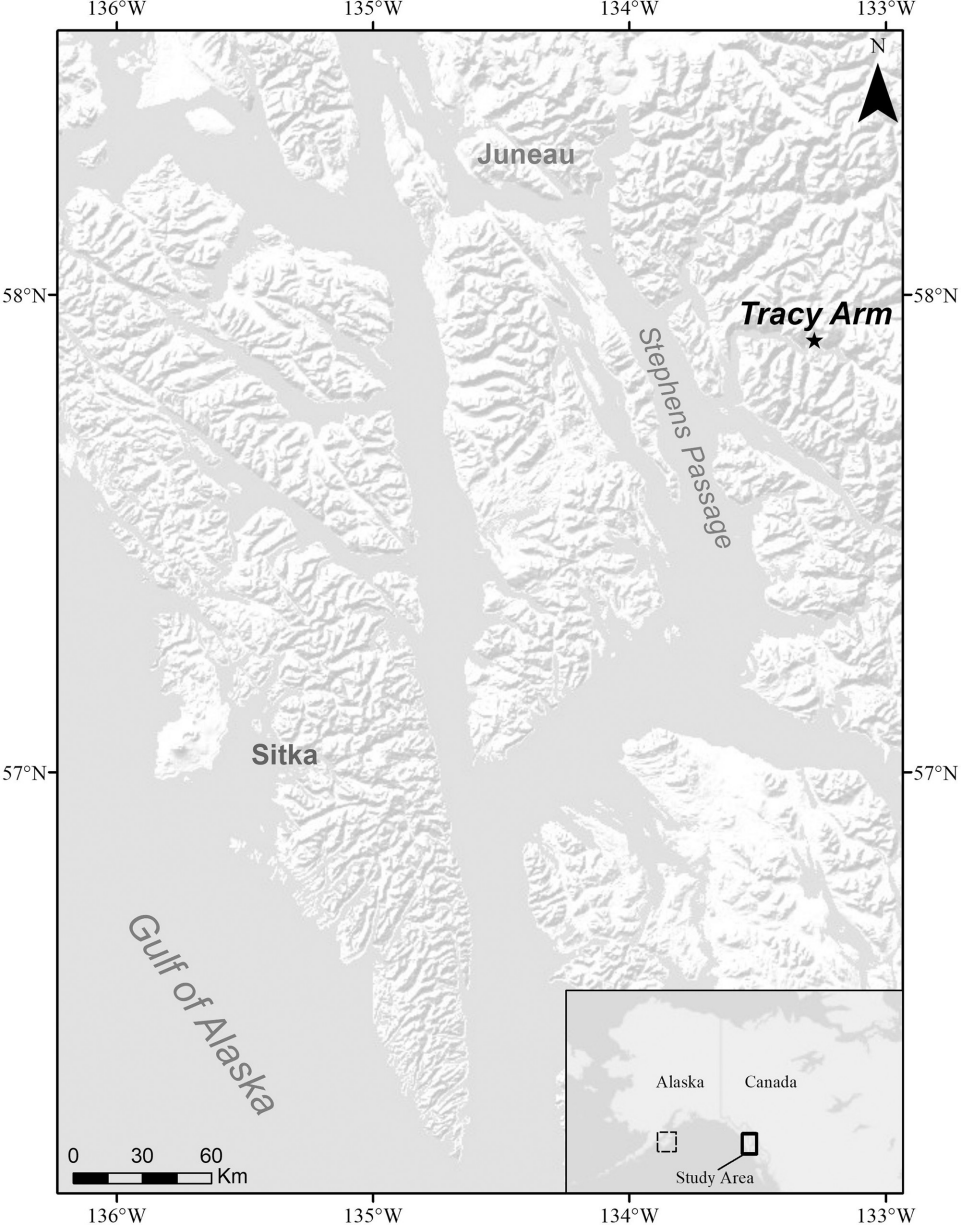
Sample collection and experimental laboratory site. The sample collection site in Tracy Arm is denoted by the star. In the inset map, the solid box indicates the zoomed in region and the dotted box marks Kodiak Island, where the laboratory experiment was completed.

Samples for this study were collected from a single site in Tracy Arm fjord, Holkham Bay in Southeast Alaska (Fig 1). The glacial fjord is 49 km long, up to 378 m deep and terminates at two tide-water glaciers, the Sawyer and the South Sawyer. Previous surveys of the fjord revealed thickets of *P*. *pacifica* as shallow as 6 m and to depths greater than 100 m [21]. The collection site was located in the central part of the fjord, 13 and 15 km from the two tide-water glaciers respectively.

**Fig 2:**
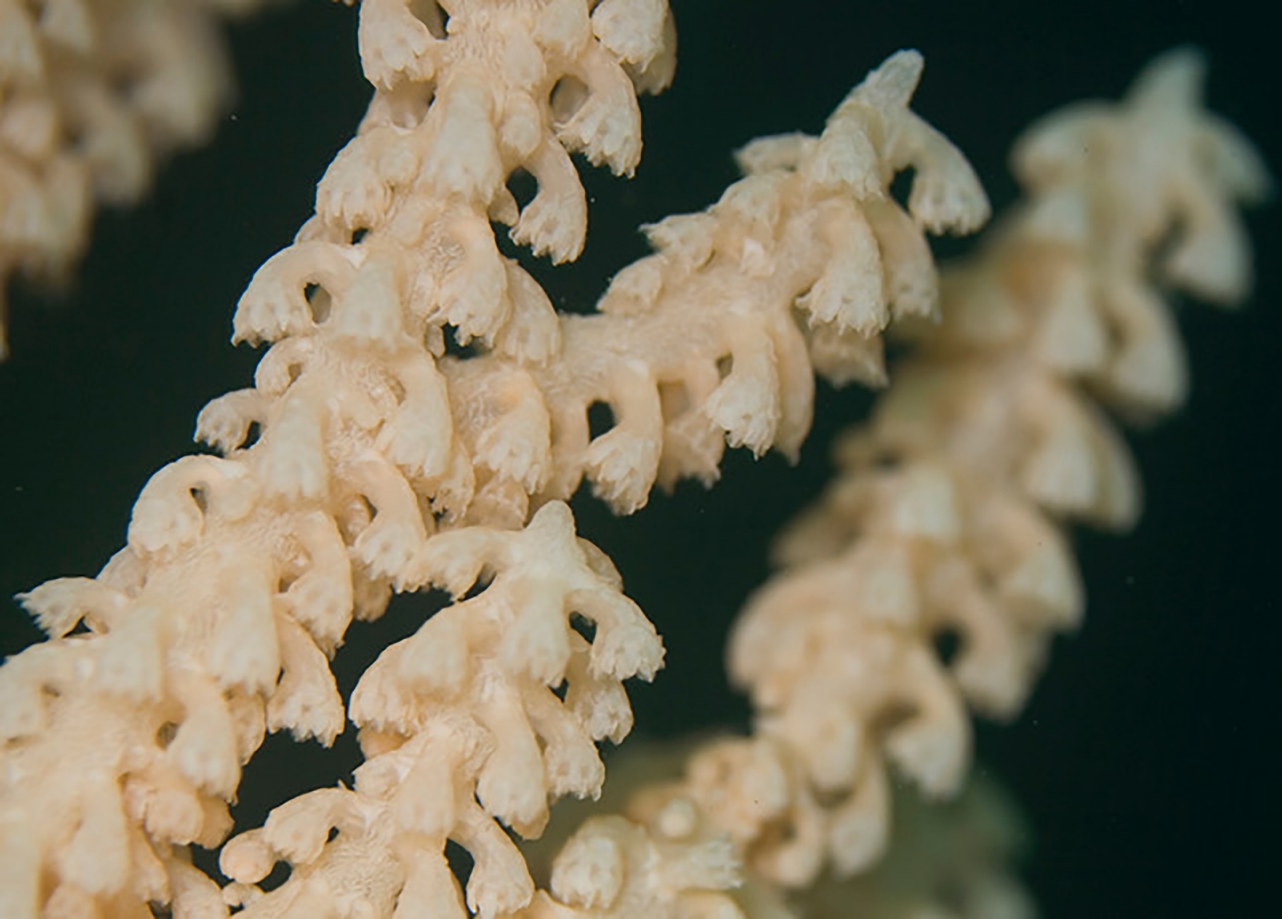
Image of growing tip on a *Primnoa pacifica* colony *in situ*.

Fifty-four *P*. *pacifica* colonies with healthy, intact growing tips were sampled with SCUBA at depths between 10–19 m from January 8–11, 2016. Colony height was measured and three sprigs, 10–15 cm long, were sampled from each colony (Fig 2). One sprig from each colony was immediately fixed in a 4% borax buffered formalin solution for 24 hours, transferred to 70% ethanol, and shipped to the Darling Marine Center (Walpole, Maine, USA) for histological processing, these were the Time 0 samples referred to later in methods. The other two sprigs were maintained live in circulating ambient seawater until they were moved to 250-ml Nalgene containers of ambient seawater, saturated with oxygen, packed on blue ice, and transported in coolers via commercial airliner to the NOAA Kodiak Laboratory (Kodiak, Alaska, USA; Fig 1). Sex was already known for more than half of the experimental colonies from previous work [21]; sex for the remaining colonies was determined during collection (by dissection) and later reconfirmed by histology in the laboratory. The colonies sampled consisted of 30 females, 20 males, and 4 were non-reproductive.

### Laboratory experiment

The design for this experiment consisted of three replicate tanks for each of two pH treatments: (1) Year 2100, which is the predicted pH at a depth of 175 m in the eastern GOA in 2100 (7.55 pH units) and (2) Control, which was the pH (7.75 pH units) for the same region in 2016. The projected Year 2100 pH was derived from the RCP8.5 (high scenario) in the Intergovernmental Panel on Climate Change (IPPC) as used in the Community Earth System Model 4 [3]; CESM4; Jessica Cross, NOAA PMEL, pers. comm.]. One sprig from each colony was randomly assigned to a treatment and then randomly assigned to a replicate within the treatment (i.e., an aquarium) in a repeated measures design. This resulted in 30 females, 20 males, and 4 non-reproductive corals represented in each treatment. The tank setup is in S1 Protocol. Treatment aquaria measured 120 cm (L) X 60 cm (W) X 60 cm (H) and unfiltered seawater was pumped into the laboratory from Trident Basin (~20.5 m depth) to a head tank and then delivered at 2 l/min to each experimental tank. pH was controlled with a monitored dosing system by bubbling CO_2_ directly into the experimental tanks of both treatments to maintain pH levels. CO_2_ input was controlled by Honeywell controllers and Durafet III pH probes controlling a gas valve. Daily pH and temperature measurements were made in each tank. pH was measured using a Ross Combination glass bulb pH electrode (Thermo Electron Corporation, Beverly, MA) calibrated with Tris buffer on the pH_F_ scale according to Millero [30,31] Once a week, water samples were collected from each tank, fixed with 0.02% mercuric chloride, and sent to the University of Alaska Ocean Acidification Research Center for alkalinity and dissolved inorganic carbon (DIC) analysis using standardized methods [32]. Those measured results were used to calculate pH, pCO2, HCO3^-^, CO3^-2^, Ω_aragonite_, and Ω_calcite_ using the “seacarb” package in R [33] (R 2.14.0, Vienna, Austria).

Sprigs were kept in total darkness to replicate their habitat conditions (Stone, Waller, pers obs). Water temperature was maintained at 4.5–5°C, which is the mean annual temperature experienced by the corals *in situ* [21]. Sprigs were suspended in the water column, tip facing downwards and tied with a Spectrafiber microfilament braided line (10-pound test). They were fed 25 ml mix of six marine microalgae (Reed Mariculture Inc., Shellfish Diet 1800) that was diluted in 450 ml of unfiltered seawater once a week, following protocol from a previous study [34]. The tanks were allowed to go static (i.e. no water flow) for 20 minutes during feeding to allow the food mixture to fully permeate the tank.

The laboratory experiment was conducted between 15 January and 22 September 2016. Throughout the duration of the experiment, no spawning or gamete release was observed. However, on 21 June (Day 158), the circulating water system of Tank 3 (Year 2100 treatment) failed, causing all but three sprigs to begin sloughing their tissue. Polyps from those sprigs were sampled immediately. The three remaining sprigs were sampled on 23 June (Day 160) and the corresponding sprigs for those colonies in the Control treatment were sampled on 29 June (Day 166) and immediately processed for histological analyses. The experiment was terminated on 22 September 2016 (Day 251) and tissues were prepared for histological processing.

### Histological processing and examination

All sprigs were assigned random numbers prior to histological processing to prevent bias. Three to nine polyps, approximately one centimeter of the 10–15 cm of sprig preserved, were dissected from each sprig for histological processing following previous protocols [21]. Polyps were sampled randomly from the sprigs as previous observations have suggested no variance in gametogenesis within colonies [21]. Polyps were decalcified with Rapid Bone Decalcifier (Electron Microscopy Sciences), then dehydrated in serial ethanol dilutions from 30% to 100%. Samples were then cleared in Toluene solution and then immersed in paraffin wax (Leica ParaPlast Plus) for approximately 48 hours at 56°C.

**Fig 3:**
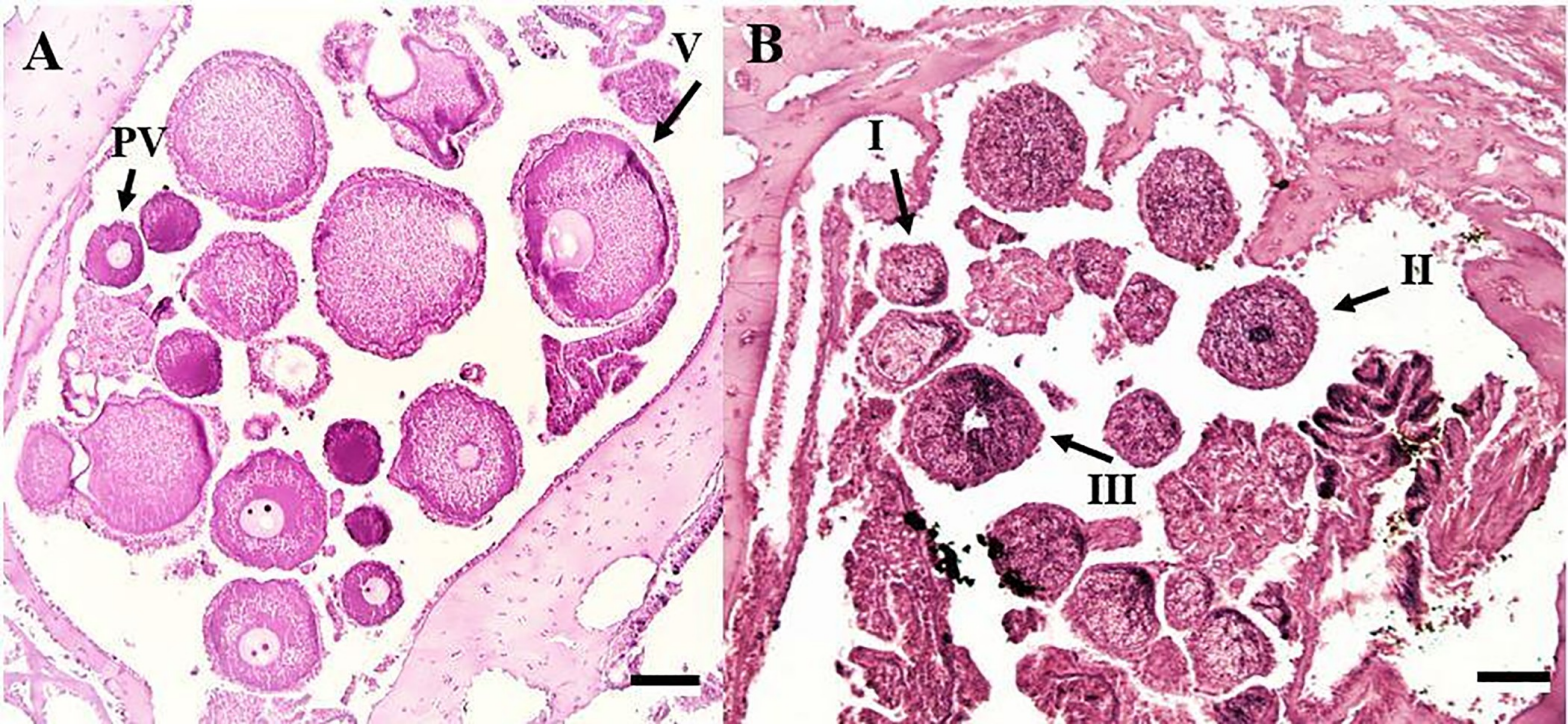
Histological sections of polyp tissue. (A) female with previtellogenic (PV) and vitellogenic (V) oocytes. (B) male with spermatocysts from three of the four stages. Scale bars represent 100 μm.

Tissue was then embedded in paraffin wax blocks and left to cool for at least 24 hours, then placed in a freezer at least one hour prior to sectioning with a microtome (Microm HM 325). All wax blocks were sliced all the way through at a consistent distance between slides to observe all gametes in the sample (i.e. serially sectioned). Specimens were sliced 6 μm thick to maintain tissue quality; the distance between serial sections was 90 μm between slides, which is the average diameter of the oocyte nucleus in *P*. *pacifica* [21]. Sectioned tissue was mounted on glass slides, dried on slide warmers, and stained with Hematoxylin and Eosin or Massons Trichrome (Fig 3).

Slides were examined using an Olympus (CX31) compound microscope with a Motic video camera attachment. Images were captured using Motic Image Plus and analyzed with ImageJ (NIH) software to calculate oocyte and nucleus diameter.

Spermatocysts were staged from I-IV, indicating increasing maturity, following the classification by Waller et al [21]. Roughly one hundred oocytes were measured for each female sprig following previous protocols to capture any variation in development stages [21]. Oocytes with a visible nucleus were the only oocytes counted to ensure there was no double counting (there is only one nucleus per oocyte). Fecundity was quantified by counting all oocytes (both previtellogenic and vitellogenic (Fig 3A) in three polyps per sprig and then averaged following Waller et al [21] to determine the average fecundity per polyp within a colony.

Histological analyses between treatments and individuals were performed using R Studio Version 3.4.1 for each study parameter. A Repeated Measures ANOVA (RMANOVA) was performed for all data comparisons to satisfy the “within subjects” assumption from the “car” package [33]. A paired t-test was performed within the same package to determine if the difference in variance between treatments was significant (p<0.05). Normality and equal variance tests were not required given the assumptions of the RMANOVA. The methods and results for the corresponding sprig analysis can be found in S1 File. Analyses were between the three treatment groups, Time Zero, Control, and Year 2100. Due to the tank failure described above, there are 18 fewer sprigs from Year 2100 in the analysis.

## Results

### Spermatogenesis

Results from the experimental treatments, Control and Time 0, are from September and June, as there was no significant difference between the two collection months for spermatogenesis. Tissue from 50 male sprigs were analyzed in this study. There were 20 sprigs from Time 0 (no experimental treatment), 18 sprigs from the Control treatment, and 12 sprigs from the Year 2100 treatment.

**Fig 4:**
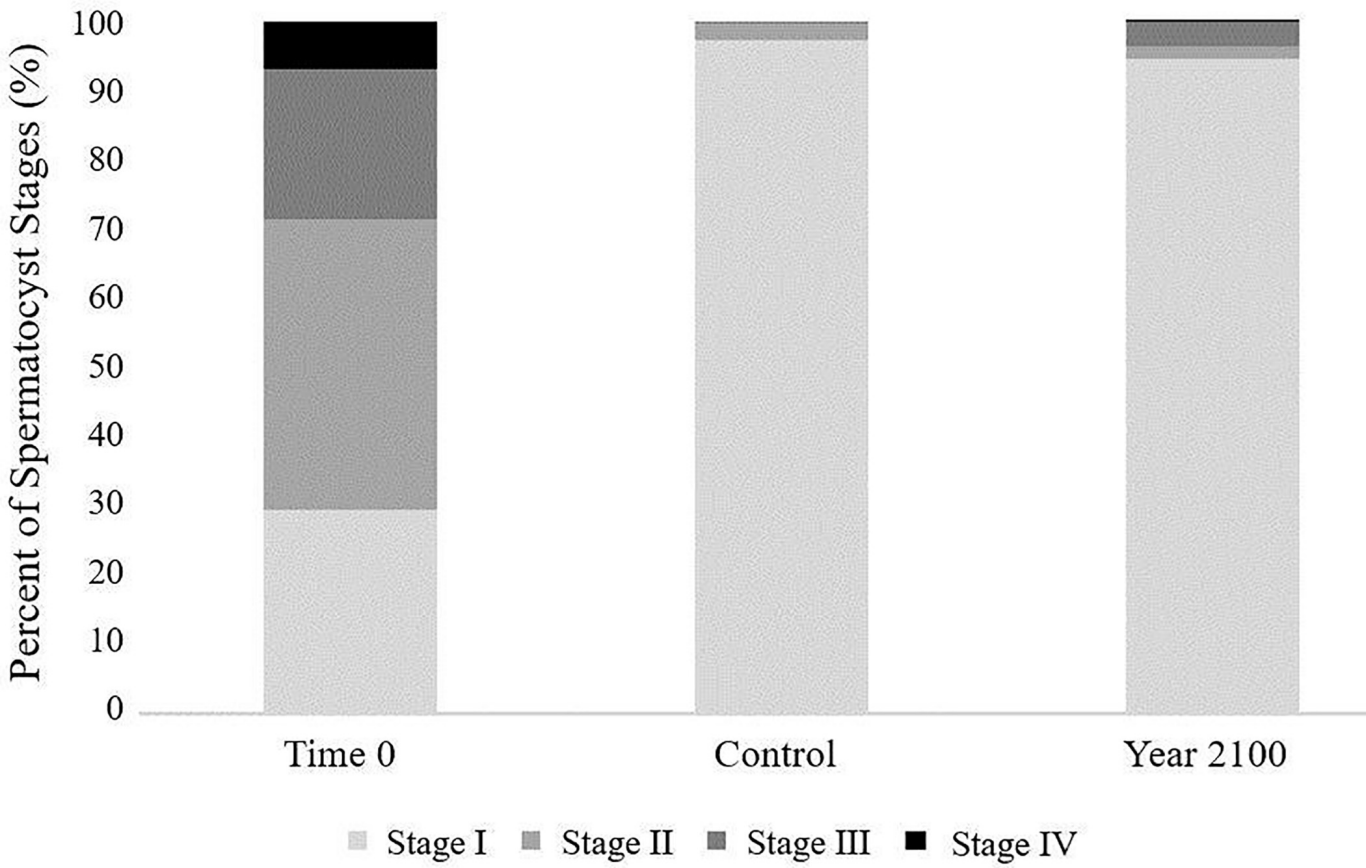
Total percent spermatocyst stage (%) versus treatment. Sperm stages increase with maturity. N indicates number of individuals and n indicates number of spermatocysts (Time 0: N = 20, n = 2000; Control: N = 18, n = 1710; Year 2100: N = 12, n = 1070).

Males from the Time 0 treatment show an average of 29.55 ± 5.15 (Standard Error) % Stage I, 41.95 ± 0.14 (SE)% Stage II, 21.7 ± 2.64 (SE)% Stage III, and 6.8 ± 1.91(SE)% Stage IV (Fig 4). Males from the Control treatment had an average of 92.39 ± 3.4(SE)% Stage I, 2.29 ± 1.01 (SE)% Stage II, 0.44 ± 0.19 (SE)% Stage III, and no Stage IV. The Year 2100 males were also primarily composed of Stage I spermatocysts, with an average of 84.33 ± 8.07 (SE)%, 1.67 ± 0.7 (SE)% Stage II, 3 ± 2.35 (SE)% Stage III, and 00.1667 ±0.13 (SE)% Stage IV.

Regardless of the spermatocyst stage, the RMANOVA showed that between the three treatments, treatment had an effect on mean percent of each ascending stage (Stage I RMANOVA: F (1,2) = 33.02, p = 9.49e-7. Stage II RMANOVA: F (1,2) = 143.32, p = 6.1e-11. Stage III RMANOVA: F (1,2) = 28.3820, p = 2.72e-06. Stage IV: F (1,2) = 8.6132, p = 2.38e-03). Post-hoc tests showed that Time 0 was statistically different from both Control and Year 2100 (Stage I p = 5.118e-09 & 1.068e-10; Stage II p = 4.728e-10 & 2.958e-04; Stage III p = 7.569e-07 & 2.968e-04; Stage IV p = 7.03e-03 & 2.48e-02), but the experimental treatments were not statistically different from one another (p = 4.34e-01; 4.34e-01, 2.92e-01; 3.43e-01).

### Oogenesis

There was a significant difference between June and September for oogenesis due to the tank failure, and so the sprigs from June have been removed from those analyses.

**Fig 5:**
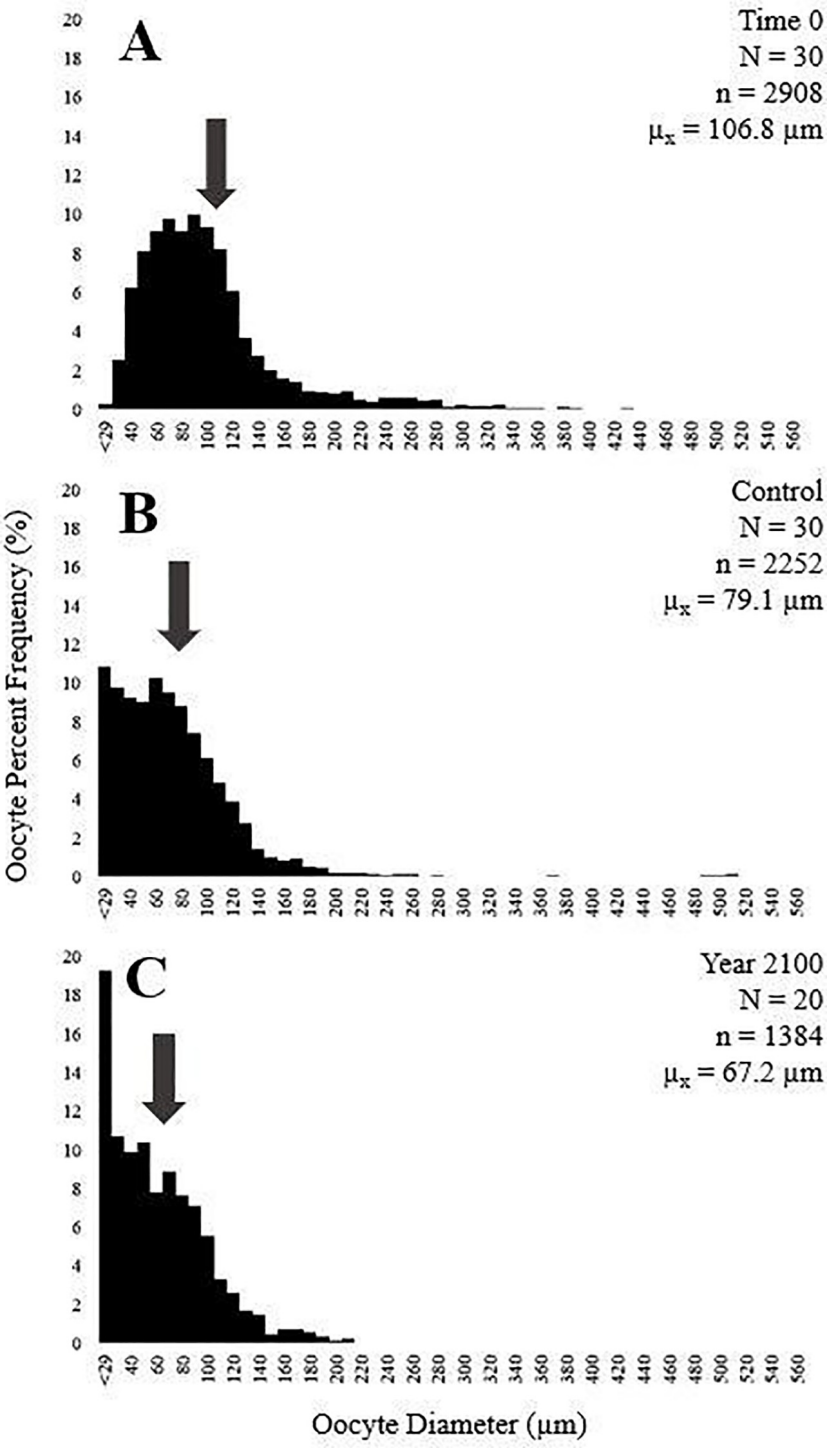
Histogram of percent oocyte frequency (%) versus oocyte diameter (μm). (A) Time 0, (B) Control, and (C) Year 2100. N indicates number of individuals, n indicates number of oocytes measured, and μ_x_ is the mean oocyte diameter (indicated by the arrows). Bins are arranged so that the minimum value within the bin is presented on the figure (i.e. 40 represents the bin range between 40–59.9 μm).

Tissue from 80 female sprigs were examined in this study, 30 from Time 0, 30 from Control and 20 from Year 2100. The mean oocyte diameter for Time 0 was 106.8 ± 1.09 (SE) μm, Control was 77.79 ± 1.12 (SE) μm and Year 2100 was 67.18 ± 1.09 (SE) μm (Fig 5). Mean oocyte diameters significantly differed among the three treatments (RMANOVA: F (1,2) = 257.11, p = <2.2e-16). Each treatment was statistically different from one another, Time 0 oocytes were significantly larger than Control and Year 2100 (p = 6.6e-16 & 6.6e-16), and Control was significantly larger than Year 2100 (p = 6.83e-04).

#### Oosorption

**Fig 6:**
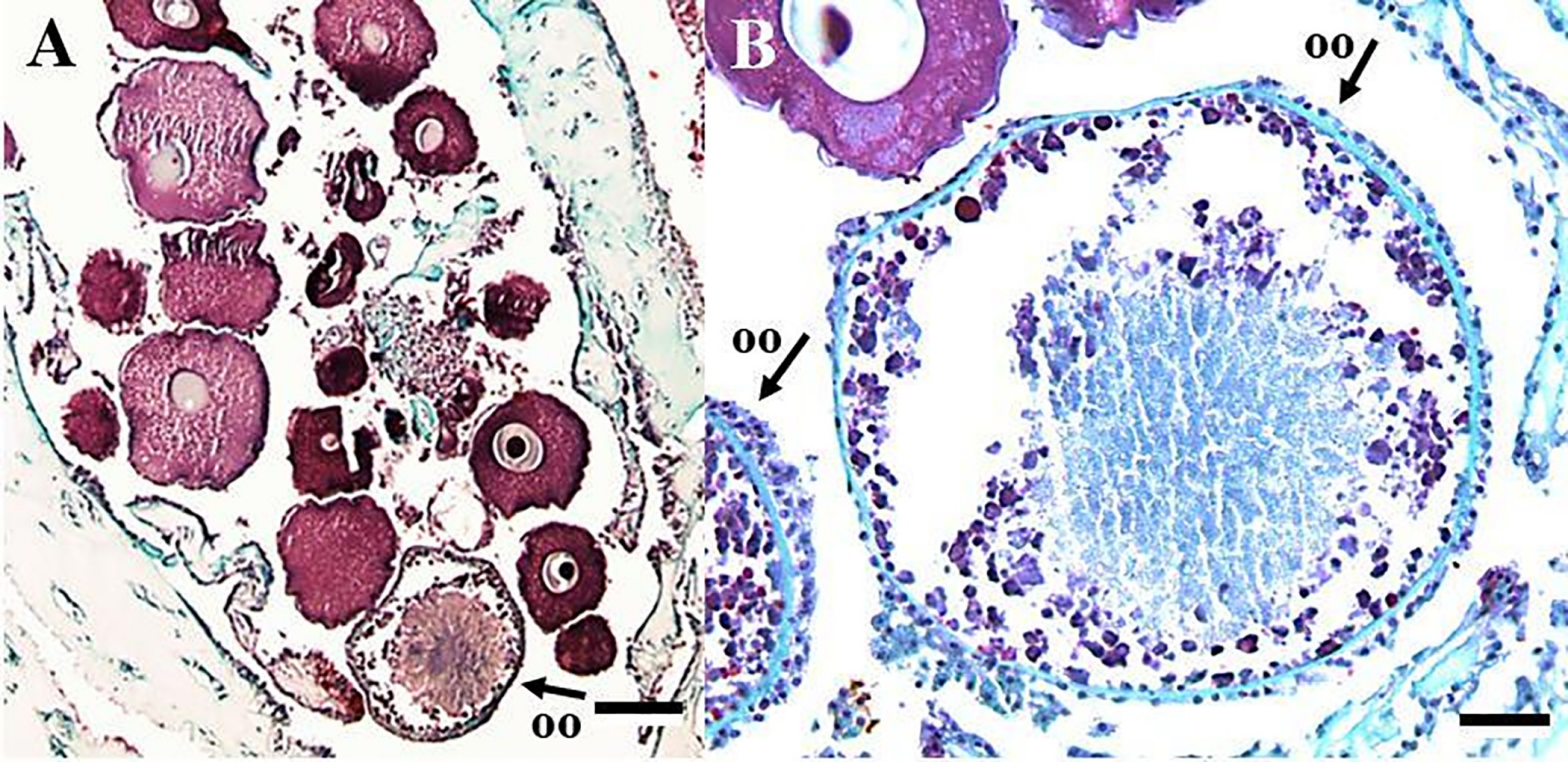
Light microscopy photograph of female polyp containing a non-nucleated oocyte. (A) shows oosorption in polyp at 4X magnification. (B) shows oosorption at 10X magnification. Scale bars are 100 μm and 500 μm respectively. OO = oocyte undergoing oosorption.

Oosorption is the process of resorbing vitellogenic oocytes to re-use the lipids for other physiological processes when under stress [13]. The lipid-dense structures (as stained by Massons Trichrome) were observed within reproductive polyps, alongside both previtellogenic and vitellogenic eggs, and generally near the gastrovascular wall. Thirty-eight of the 80 female sprigs (48%) had structures measuring ~220–802 μm (the same size as vitellogenic oocytes) near oocytes which did not have a nucleus and were composed of lipid-dense concentrations (Fig 6). Twenty percent of the Time 0 female sprigs, 63% of the Control female sprigs, and 65% of the Year 2100 female sprigs had these structures. The number of actual oocytes undergoing oosorption per female could not be counted due to methodology constraints (serial sectioning distances were designed to count viable oocytes only), but is an interesting avenue of future study using a combination of histology and Transmission Electron Microscopy.

There was no apparent relationship between the presence of cells undergoing oosorption and treatment type nor was the presence of the structures at Time 0 an apparent indicator for presence in Control or Year 2100 sprigs from the same colony. To determine this, the authors compared the sprigs from all three treatments to one another.

### Fecundity

**Fig 7:**
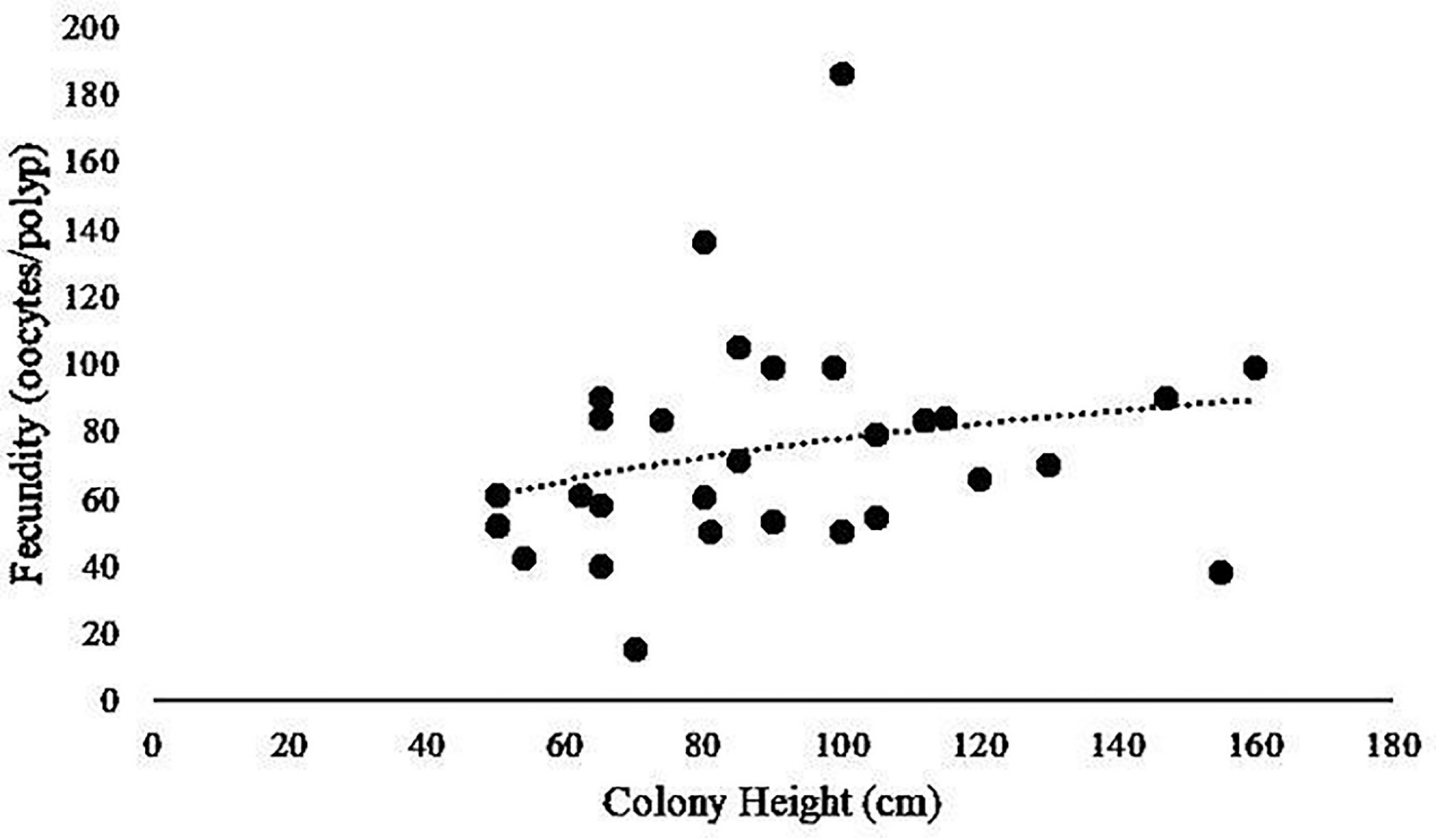
Maximum fecundity (oocytes per polyp) versus colony height. Trend line is logarithmic. R^2^ value of 0.057.

All reproductive females had a maximum colony height between 42 and 160 cm and maximum fecundity was positively related to colony height, albeit with a low coefficient of determination, which is not statistically significant (R^2^ = 0.057; Fig 7).

**Fig 8:**
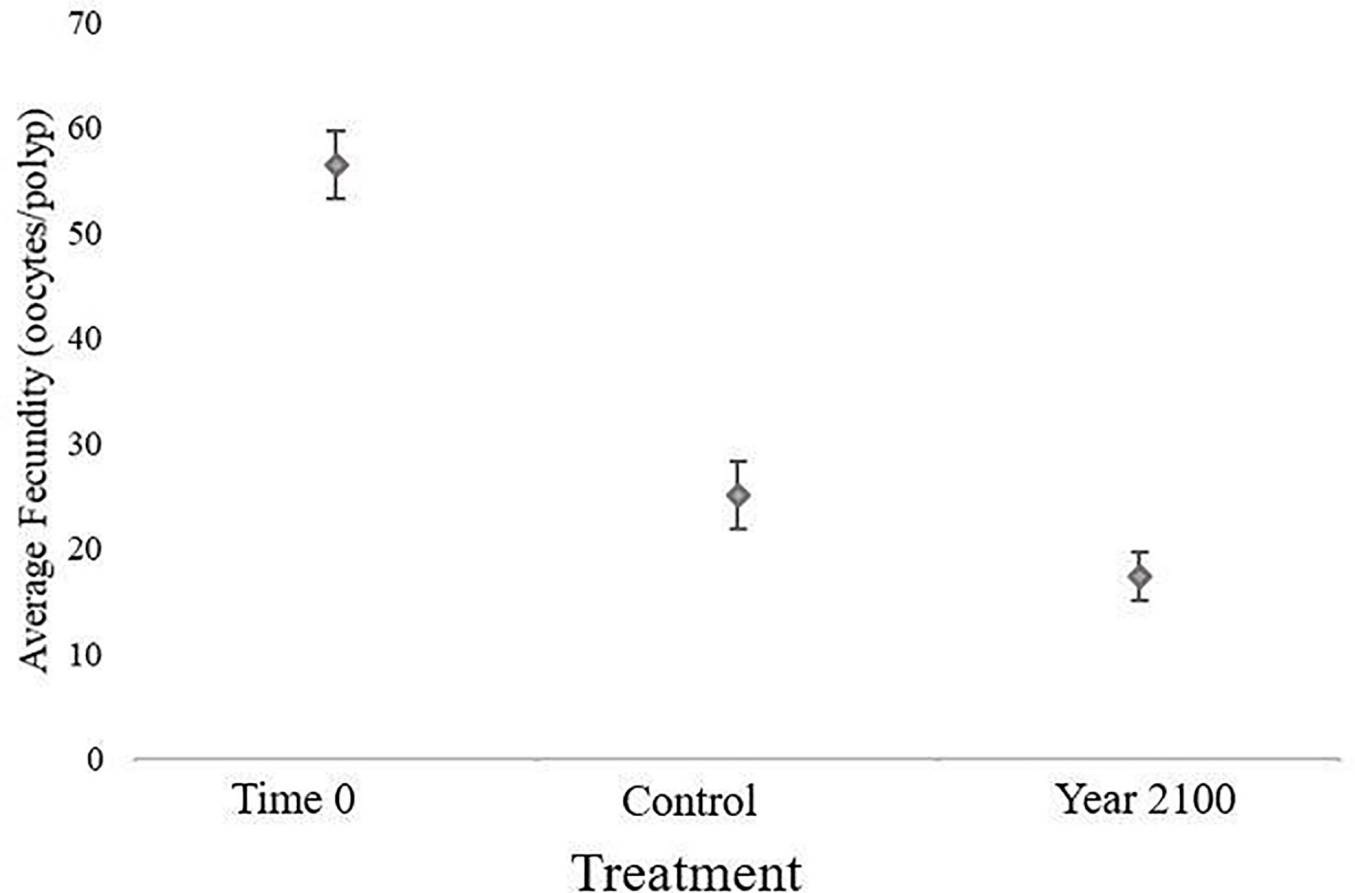
Average fecundity (oocytes per polyp) for sprigs versus treatment. Error bars indicate standard error. N represents number of individuals (Time 0: N = 30; Control: N = 30; Year 2100: N = 20).

The average fecundity for Time 0 was 56.43 ± 3.13 (SE) oocytes per polyp, Control was 25.1 ± 3.19 (SE) oocytes per polyp, and Year 2100 was 17.32 ± 2.24 (SE) oocytes per polyp (Fig 8). This is total oocytes, not split between previtellogenic and vitellogenic oocytes. Treatment had an effect on average fecundity (RMANOVA: F (1,6) = 6.027, p = 9.36e-05), and each treatment was statistically different from the Time 0 (Time 0: Control p = 1.91e-11; Time 0: Year 2100 p = 9.97e-09). Year 2100 was not significantly different from Control (p = 0.6309), however, Year 2100 fecundities are 31% smaller than the Control.

## Discussion

Comparisons between this dataset and the previous work from the same coral population [21] are in S1 Information. The sprigs from the Control and Year 2100 treatments were significantly different than the Time 0 samples. The Control and Year 2100 results were statistically the same for the spermatocyst stages, oocyte diameter was statistically different among all treatments; while the fecundity was not statistically different between Control and Year 2100, but was 31% lower. This negative trend in fecundity and oocyte development could be attributed to the animals being stressed by being in the tanks, regardless of pH treatment, and likely reflects the absence of a homogeneity between the tank environment and the corals’ natural habitat [35].

The significant differences observed between the Time 0 samples and the experimental treatments were probably influenced by artifacts associated with the laboratory conditions. Though these errant artifacts did cause significant differences in oocyte diameter and fecundity; effects from the pH treatment can still be discerned. Females in the Year 2100 treatment had a smaller average oocyte size and lower fecundity than those in the Control treatment. The Year 2100 females also had the highest proportion of reabsorbing oocytes compared to Control and Time 0. Considering the developmental differences between the pH treatments, it appears unlikely that these females would spawn vitellogenic oocytes (or spawn usual numbers of vitellogenic oocytes) under pH conditions predicted for Year 2100, at least as an acute response. There is also potential oocytes could be too small to fertilize successfully, as studies on other marine invertebrates have shown larger oocytes influence collision frequency with sperm and increased fertilization rates [36]. Even if viable larvae were produced, in this species fecundities may also be too low for effective recruitment events needed to maintain whole populations.

### Spermatogenesis

While there was no significant difference between Control and Year 2100 treatments with regards to spermatocyst stages, the Year 2100 spermatocysts (Stage IV) were much more developed than those from the Control (Stage I), which is an interesting trend. Previous studies [37] have shown no statistical difference in sperm stages in the scleractinian coral, *Leptopsammia pruvoti*, though in that study sperm were equally developed in pH 7.4 and in pH 8.07, unlike *P*. *pacifica* which were trending 3 stages lower in the Control treatment. If sperm are able to develop faster under lower pH conditions, as this trend suggests, it may have implications for sperm condition. Caldwell and associates [38] performed a study looking at sperm swimming speeds in sea urchins at different pH ranges and temperatures. The lower pH treatments found a higher mean percent motility and swimming velocity. It was also noted, however, that while those increased, it would come at a metabolic cost, indicating a decrease in sperm longevity [38]. The authors concluded that ocean acidification has a potential to disrupt the reproductive and development processes and should still be considered a primary concern [38]. Any improved potential increase in development time and sperm condition for these corals, could therefore be cancelled out by reduced oocyte fitness, fertilization success, or decreased overall embryonic condition [38,39].

### Oogenesis

While differences in oocyte diameter between Time 0 and the other treatments indicate the shift in seasonality and laboratory effect, the difference between the Control and Year 2100 may indicate a pH effect. Most notably, there were no vitellogenic oocytes in the Year 2100 treatment (a state not seen in the natural population [21], likely owing to the lipid and protein content not fully developing and thus decreasing maturation through vitellogenesis. During vitellogenesis, the lipids within the oocyte are concentrated and the oocyte roughly doubles in size, increasing the energy store for the future larva [40]. If these smaller oocytes were spawned and successfully fertilized, they may have insufficient lipid reserves, which could decrease the larval longevity, fitness and may increase the risk of predation [36]. For example, smaller eggs in sea urchins under decreased pH conditions can have less lipid content [39,41] with negative consequences for larval fitness such as increased age at metamorphosis and reduced fitness by smaller larval size or morphological abnormalities of newly settled juveniles [41]. Larval longevity is also dependent on high lipid content of oocytes in some reef building scleractinian corals [42–45]. A reduced larval longevity due to decreased lipid reserves could decrease the dispersal or cause larvae to settle in less suitable environments to conserve energy for metamorphosis. Recently settled juveniles require food-fall from the surface layers [46] and spawning and gametogenesis may be timed with surface water productivity [21,46]. If this timing is disrupted or the larvae settle to a region without this food fall, growth and survival may be limited.

#### Oosorption

Initially, we considered the structures to be unidentified extracellular material, however they are more consistent with reabsorbed oocytes when compared with previously published observations [13]. These structures had a membrane surrounding the mass of cells, similar to the vitelline envelope in oocytes (Fig 6). Small cells surrounding the membrane appeared to have suspended nuclei which were connected by a less rigid conglomerate, similar to previous observations of intracellular gaps leading to an unorganized association of cells on the exterior of the oocyte [13]. Small, round concentrations of cells within the membrane stained as lipids, as oocytes do, however, they were more densely packed and unorganized compared to those in vitellogenic oocytes.

The combined results of smaller average oocyte diameters for Year 2100 compared to Control treatments (67.2 μm and 77.79 μm, respectively) and an increased presence of large, non-nucleated lipid-dense structures (65% and 63%) suggest that lipid reserves may have been redirected from gametogenesis to other metabolic processes under lower pH conditions and lab conditions, however it was not within the scope of this study to determine this conclusively. This difference in percentage alone may not seem significant, however the increased occurrence of oosorption coinciding with smaller oocytes could indicate that spawning of fully developed oocytes (i.e. natural spawning) may be affected by acidified conditions.

Based on this study, fecundity and oocyte size may not be related to the presence of oosorption, so oosorption does not prevent other oocytes from developing within the same individual, and smaller eggs are likely held in reserve to develop from the reabsorbed lipids [13]. The process likely occurs naturally since these structures were observed in females from all treatment groups; and may not be unique to *P*. *pacifica* or gorgonians. For example, the scleractinian coral *Acropora millepora* uses lipid reserves when under stress to provide energy to maintain net calcification rates [10].

The increase in oosorption rate in experimental treatments compared to Day 0 likely indicates a stress response of the corals to laboratory and to a lesser degree low pH conditions. Oosorption has been indicated as a stress response to adverse holding conditions including inadequate food supply or poor quality, stagnant water supply, and varying water temperatures in other phyla [13]. Since the sprigs were fed regularly and kept under normal temperatures, the stress some ambiguous aspect of the laboratory conditions likely induced the increased rate of resorption. Species without significant nutrient reserves that devote a large portion of their energy budget to gametogenesis may also reabsorb gametes in response to starvation or stressors that induce a rapid energy deficit [13]. Further studies should be done on cold-water coral species to determine if this is the purpose of oosorption, and if so, where those energy reserves are being redirected.

### Fecundity

The fecundity and height relationship from this study corroborates previous results for *P*. *pacifica* that female colonies with total height greater than 50 cm are reproductive [21], and that there is a slightly positive relationship with size and fecundity as shown by the logarithmic line (Fig 7). Age and size at sexual maturity has not been studied for this population, but could be an interesting future study to investigate current population growth patterns and how those compare under acidified conditions.

While all three fecundities are different from one another, the Year 2100 average fecundity is 31%lower than the Control treatment. As fecundity can be used as a proxy for reproductive effort of a colony [47], this marked decrease in fecundity with an increase in acidity elucidates a decrease in reproductive effort under stress. This reduced effort could cause a reproductive bottleneck by indicating a response to an energy imbalance within the corals. If they cannot put as much energy towards reproduction to keep up with essential life processes during stress (i.e. OA, warming, hypoxia; [11]) then the population may decline while growth or calcification appear unchanged.

## Conclusions

The results from this study provide preliminary data on the effects of OA on the processes of gametogenesis and reproduction for cold-water corals. While the spermatogenesis results cannot be fully compared due to the observed laboratory effect, the oogenesis results are notable. The smaller oocytes and lower fecundity from the Year 2100 females indicates an inability to fully allocate resources to oogenesis in acidified conditions. These results combined with the increased presence of oosorption could have potentially deleterious effects for red tree coral populations in future oceans if OA continues at projected levels.

The apparent laboratory effect observed in this study indicates a need to better understand the natural environment of cold-water corals, particularly gorgonians, to more accurately replicate *in situ* conditions in laboratory experiments. This could also potentially be amended by adding a longer acclimation period prior to experimentation and future experiments on similar species should account for a longer period prior to pCO_2_ manipulations to attempt to prevent these effects. Though our goal was to conduct the experiment for one year, or a full reproductive cycle, we were unable to maintain the corals for more than 200 days. We recommend that future experiments with red tree corals be limited to 200 days to avoid the deleterious physiological effects of residing in the laboratory or alternatively that *in situ* free-ocean CO_2_ enrichment experiments be used [48].

While commercially important species have been the primary focus of OA studies to date, there is a need to understand the effects of OA on other species that play other important roles in marine ecosystems. Keystone and foundation species that create habitat and provide structure for commercially important species need to be included in the portfolio of this emerging area of research and should include multiple life history stages to understand potential carry-over effects from generation to generation.

## Supporting information

S1 Protocol Information detailing how the corals were arranged in the tanks and protocol for their care for the duration of the OA experiment. (DOCX)

S1 File Methods and results of the sprig analysis. (DOCX)

S1 Information The OA study was compared to the 2014 reproductive study by Waller and associates. (DOCX)
