# Supplementary material for: The effects of in-vitro pH decrease on the gametogenesis of the red tree coral, Primnoa pacifica
Source: PLoS One. 2019 Apr 18;14(4):e0203976. doi: 10.1371/journal.pone.0203976 (PMC6472723; doi:10.1371/journal.pone.0203976)
Supplement: S1 Protocol — (DOCX) [file pone.0203976.s001.docx]

**Supplementary Protocol1**

All information regarding the tank experiment was provided by Allison Batemen, Fisheries Biologist and Seawater Laboratory Coordinator at the Kodiak Fisheries Research Center.


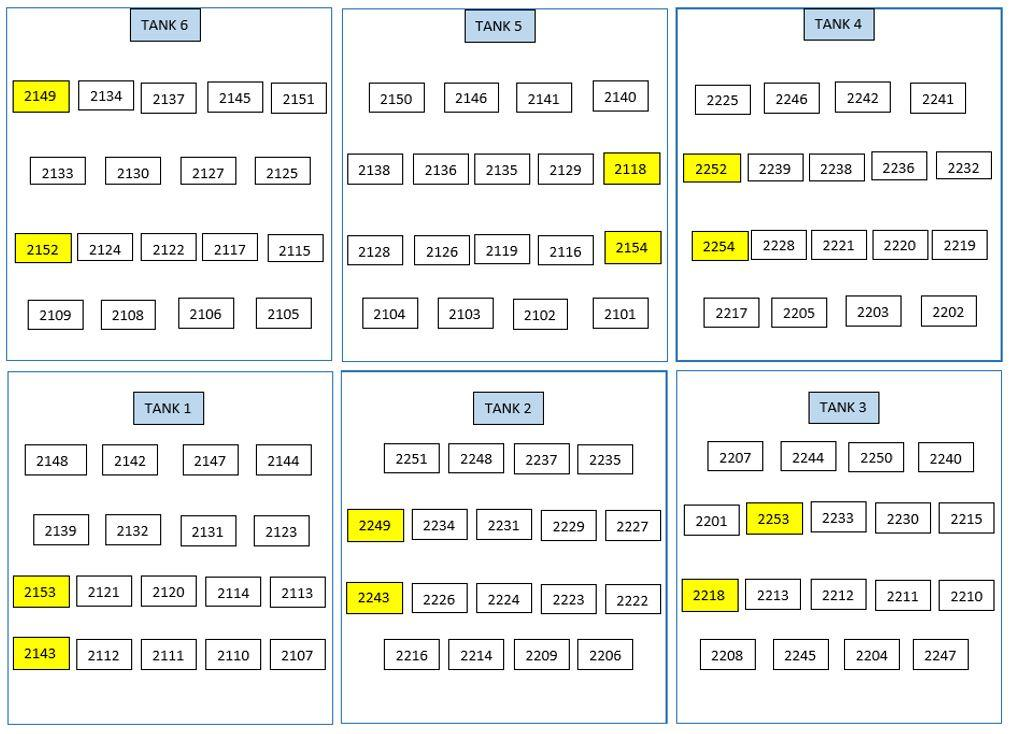


**Figure A.** Tank setup at Kodiak Fisheries Research Center, AK. Tanks 1, 5, and 6 were treatment A and tanks 2, 3, and 4 were treatment B. Each tank held 18 sprigs split between four rows. Highlighted cells represent individuals that were “spares” as their sex could not be determined to absolute certainty on the cruise.

Sprigs were suspended in the water column, tip facing downwards and tied with a Spectrafiber microfilament braided line (10-pound test). They were fed 25 ml of a unique mixture of six marine microalgae (Reed Mariculture Inc., Shellfish Diet 1800) that was diluted in 450 ml of unfiltered seawater once a week. The mixture was slowly and evenly poured throughout each tank, and the main water line was removed to ensure the food was not flushed out. The food would take approximately an hour and a half to completely drain from the tank once the water line was put back in. Any remaining food bits or sediment were siphoned away every 10 days to avoid disturbing the corals but also maintain a clean flow-through.

Water temperature was maintained at 4.5 - 5^◦^C in continuously circulating seawater (2 l/min) following the same exposures used for recent acidification experiments including Alaskan crabs and fishes [SP1]. Each day, the pH and temperature were measured in each of the tanks. If a tank’s pH differed by .02 pH units, the set temperature was readjusted. If the temperature of a tank was out of range, the flowrate within that tank was readjusted, if any of the tanks were out of range by 0.2℃, the flowrates for each tank was rechecked and adjusted. Once a week, water samples were taken for alkalinity and dissolved inorganic carbon (DIC) analysis.

**Supporting Protocol References**

1. Long WC, Swiney KM, Harris C, Page HN, Foy RJ. Effects of ocean acidification of juvenile red king crab (*Paralithodes camtschaticus)* and Tanner crab (*Chionoecetes bairdi*) growth, condition, calcification, and survival. PLoS ONE. 2013;8: e60959.
