# Supplementary material for: The effects of in-vitro pH decrease on the gametogenesis of the red tree coral, Primnoa pacifica
Source: PLoS One. 2019 Apr 18;14(4):e0203976. doi: 10.1371/journal.pone.0203976 (PMC6472723; doi:10.1371/journal.pone.0203976)
Supplement: S1 File — (DOCX) [file pone.0203976.s002.docx]

**Supplementary File 1**

**Methods**

Volumetric measurements were done at the beginning and end of the experiment at Kodiak Fisheries Research Center. No volumetric data were collected on June 21, 2016 on the failed tank since the tissue was fragile and preserved immediately so as to not cause further desiccation or damage. Pictures were also taken of each individual sprig before and after the tank experiment to determine any difference in the tissue in response to the tank conditions. Comparisons were made between the Time 0 and September images, excluding the sprigs from the failed tank. Length and surface areas of live tissue were measured three times per sprig using ImageJ software. Volumetrics, lengths and surface areas were compared between the Ambient and 2100 treatments to determine if there was a significant difference between treatments. Analysis was done in R Studio, using the “car” package.

**Results and Discussion**

**Table A.** Average results from exterior sprig measurements. For all measurement types and sampling dates, the number of sprigs was 35. SE indicates standard error.


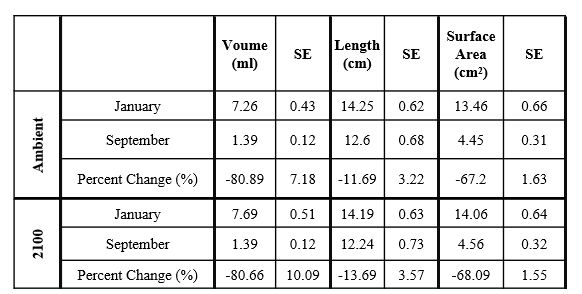


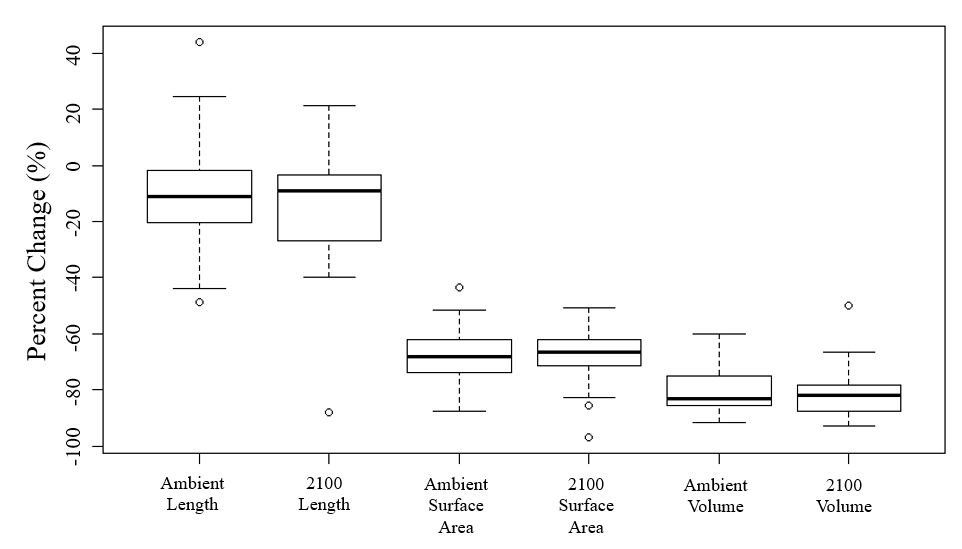


**Figure A**. Boxplot distribution of percent change (%) versus treatment and measurement type. N represents number of individuals. For each treatment and methodology, N = 35.
