# Supplementary material for: The effects of in-vitro pH decrease on the gametogenesis of the red tree coral, Primnoa pacifica
Source: PLoS One. 2019 Apr 18;14(4):e0203976. doi: 10.1371/journal.pone.0203976 (PMC6472723; doi:10.1371/journal.pone.0203976)
Supplement: S1 Information — (DOCX) [file pone.0203976.s003.docx]

**Supplementary Information 1**

The OA dataset was split between end month, while this did result in a change of average oocyte diameter and fecundity, the significance was the same. For the purposes of the paper, the dataset remained unsplit, however, the results from the two end months are provided here. The OA dataset was also compared to the 2014 reproductive dataset. When compared to the 2014 reproductive dataset, all the tank samples were statistically different from previous years and were significantly lower.

**Results and Discussion**





**Figure A.** Mean percent spermatocyst stage (%) versus treatment including the 2014 reproductive dataset. Sperm stages increase with maturity. N indicates number of individuals and n indicates number of spermatocysts (September 2010: N = 15, n = 1621; June 2011: N = 15, n = 1600; September 2011: N = 8, n = 766; January 2012: N = 14, n = 895; January 2013: N = 15, n = 1149; Time 0: N = 20, n = 2000; Ambient: N = 18, n = 1710; 2100: N = 12, n = 1070).


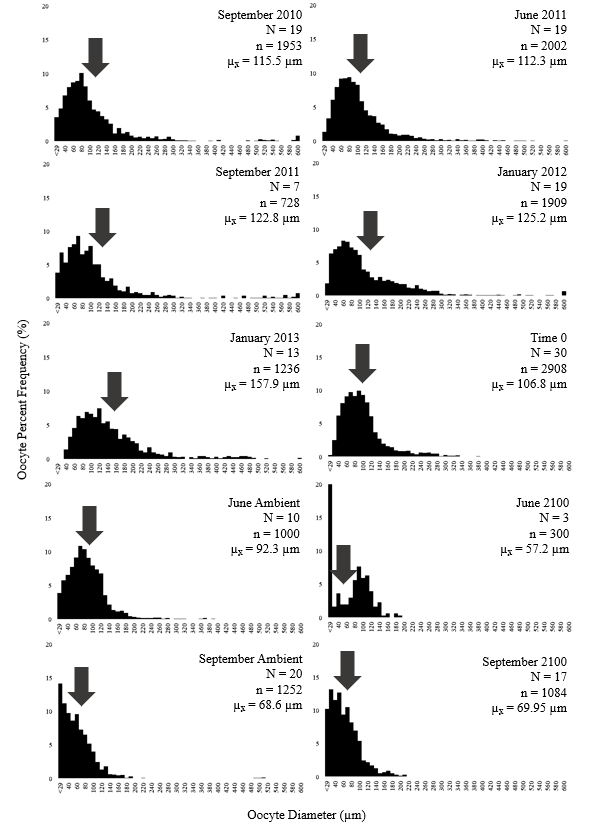


**Figure B.** Percent oocyte frequency (%) versus oocyte diameter (µm) by treatment and month. From top to bottom and left to right: September 2010, June 2011, September 2011, January 2012, January 2013, Time 0, Ambient, and 2100. N indicates number of individuals, n indicates number of oocytes measured, and µ_x_ is the mean, shown by the arrows.

**Table A.** End of experiment average oocyte diameter split by collection month. SE indicates standard error.


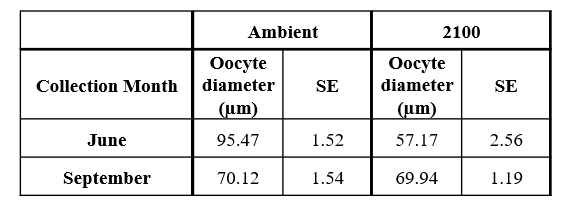


**Table B.** Average oocyte diameters for 2014 reproductive dataset. SE indicates standard error.


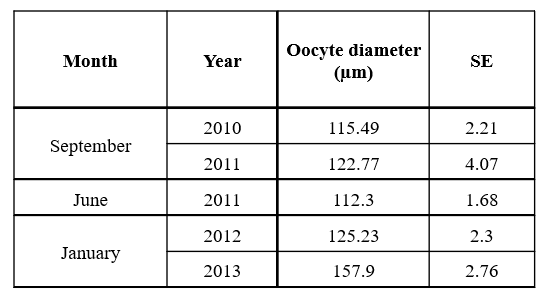


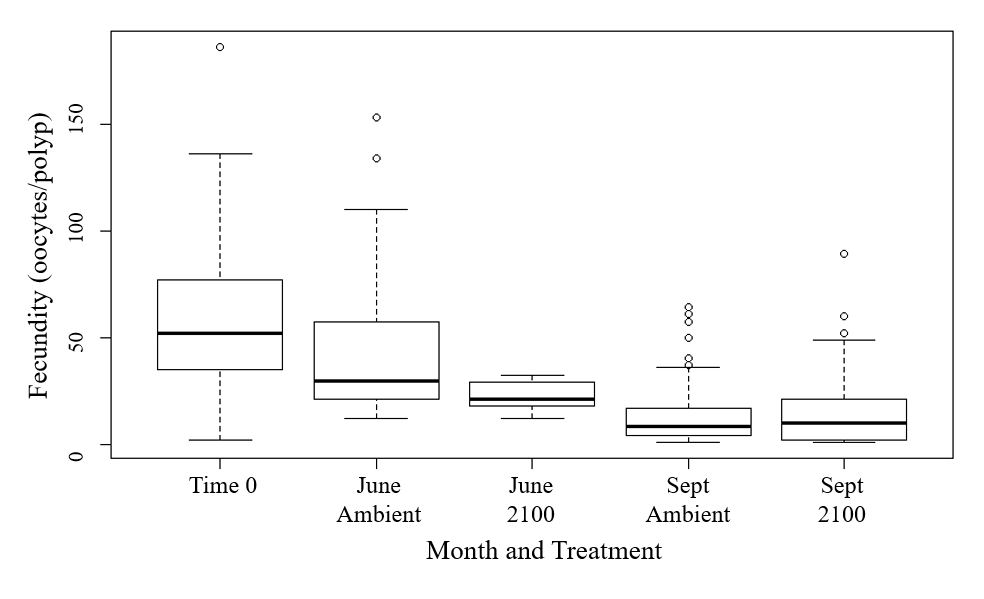


**Figure C.** Boxplot distribution of fecundity (oocytes per polyp) versus treatment, separated by collection month. N indicates number of individuals (Time 0: N = 29; June Ambient: N = 10; June 2100: N = 3; September Ambient: N = 20; September 2100: N = 17).

**Table C.** Average fecundity (oocytes per polyp) for 2014 reproductive dataset. SE indicates standard error.


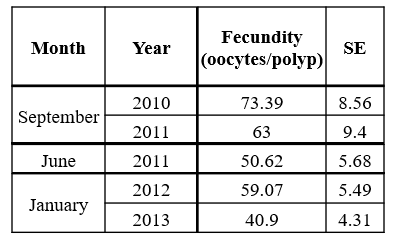


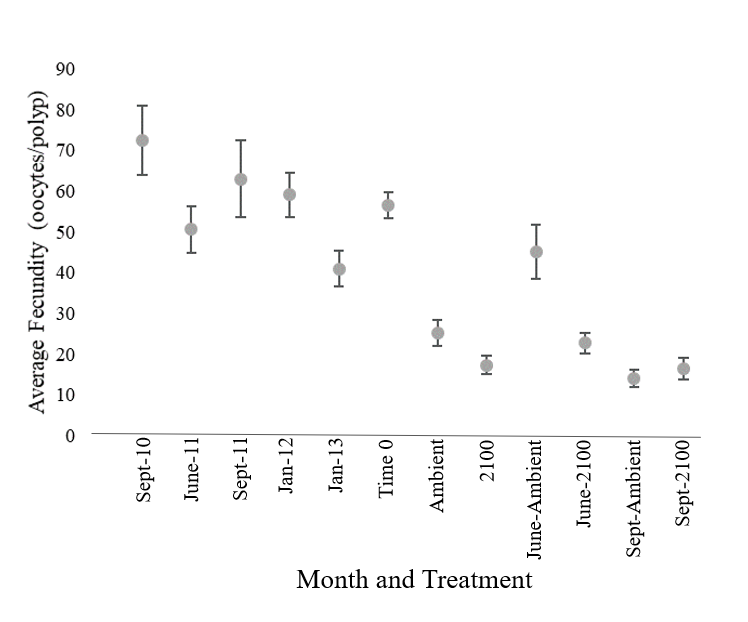


**Figure D.** Average fecundity (oocytes per polyp) versus treatment, including the 2014 reproductive dataset. Error bars represent standard error. N indicates number of individuals (September 2010: N = 19; June 2011: N = 19; September 2011: N = 7; January 2012: N = 19; January 2013: N = 13; Time 0: N = 29; Ambient: N = 30; 2100: N = 20; June Ambient: N = 10; June 2100: N = 3; September Ambient: N = 20; September 2100: N = 17).
